# AHCC^®^, a Standardized Extract of Cultured Lentinula Edodes Mycelia, Promotes the Anti-Tumor Effect of Dual Immune Checkpoint Blockade Effect in Murine Colon Cancer

**DOI:** 10.3389/fimmu.2022.875872

**Published:** 2022-04-20

**Authors:** Hong-Jai Park, Sunjin Boo, Inkeun Park, Min Sun Shin, Tsukasa Takahashi, Jun Takanari, Kohei Homma, Insoo Kang

**Affiliations:** ^1^Department of Internal Medicine, Yale University School of Medicine, New Haven, CT, United States; ^2^Department of Internal Medicine, Jeju National University School of Medicine, Jeju, South Korea; ^3^Department of Internal Medicine, Gachon University Gil Medical Center, Incheon, South Korea; ^4^Research and Development Division, Amino Up Co., Ltd, Sapporo, Japan

**Keywords:** AHCC^®^, immunotherapy, MC38 tumor, microbiome, tumor-infiltrating lymphocytes

## Abstract

Treatment strategies combining immune checkpoint blockade (ICB) with other agents have emerged as a promising approach in the treatment of cancers. AHCC^®^, a standardized extract of cultured *Lentinula edodes* mycelia, has been reported to inhibit tumor growth and enhance immune cell function. Here we investigated whether AHCC^®^ promotes the therapeutic effect of immunotherapy in cancers. A combination of oral AHCC^®^ and dual immune checkpoint blockade (DICB), including PD-1/CTLA-4 blockade, had reduced tumor growth and increased granzyme B and Ki-67 expression by tumor-infiltrating CD8^+^ T cells in MC38 colon cancer bearing mice compared to a combination of water and DICB. In the same tumor bearing mice, AHCC^®^ and DICB treatment also altered the composition of the gut microbiome with the increased abundance of the species of *Ruminococcaceae* family which is associated with increased therapeutic efficacy of immunotherapy. The anti-tumor effect of AHCC^®^ and DICB was not found in MC38 tumor-bearing mice treated with antibiotics. These data suggest that AHCC^®^ increases the anti-tumor effect of DICB by enhancing T cell function and affecting the gut microbiome.

## Introduction

The full activation of naïve T cells into effector T cells requires three signals that include T cell receptor (TCR) activation by antigenic peptide, cytokine signals, and co-stimulation *via* co-stimulatory molecules (1). This event can be regulated by immune checkpoint molecules which are important for maintaining tolerance against self. Among these molecules, the best-known are programmed cell death protein 1 (PD-1) and cytotoxic T lymphocyte-associated antigen 4 (CTLA-4) which regulate T cell receptor signaling and activation *via* binding to the ligands PD-L1/PD-L2 and CD80/CD86, respectively. Activated T cells rapidly upregulate PD-1 and CTLA-4 to negatively regulate T cell activation. Blocking the binding of these molecules to the ligands can lead to increased T cell activation, proliferation and cytotoxicity. Indeed, immune checkpoint blockade (ICB) therapy targeting PD-1 and CTLA-4 pathways, often in combination as dual immune checkpoint blockade (DICB) therapy, has revolutionized the treatments of cancers including melanoma, non-small cell lung cancer, and bladder cancer (2, 3). A combination of ipilimumab (anti-CTLA-4 antibodies or Abs) and nivolumab (anti-PD-1 Abs) was approved by the FDA for the treatment of patients with metastatic colorectal cancer who have been treated previously with standard chemotherapy drugs (4). However, a number of cancers do not respond or become resistant to ICB therapy while patients treated with ICB often develop immune-related adverse events such as pneumonitis, colitis and endocrinopathies, which can be serious and fatal (2).

For many years natural products such as edible mushrooms have been considered as possible resources for treating medical conditions including cancers although therapeutic benefits and mechanisms of natural products are yet to be elucidated (5). AHCC^®^ is a standardized extract of cultured *Lentinula edodes* mycelia, which comprises oligosaccharides, amino acids and minerals (6). Mushroom extracts, especially polysaccharides, are reported to enhance immune responses to tumors by affecting T and other immune cells (7). The most abundant component of AHCC^®^ is oligosaccharides which comprise about 74% of the dry weight of AHCC^®^ (6). Previous human and animal studies showed the possible effects of AHCC^®^ on the frequency and function of T cells (8). Also, the suppression of tumor growth by AHCC^®^ was reported in murine models of melanoma and hepatoma (9, 10). However, little is known whether AHCC^®^ can promote the tumor suppressive effect of ICB therapy.

Here we studied the additive effect of AHCC^®^ to DICB in suppressing tumor growth using mice bearing murine colon cancer. We also explored the possible mechanisms of such an additive effect of AHCC^®^ by analyzing tumor infiltrating and splenic T cells as well as gut microbiome in the same mice. The results of our study showed that AHCC^®^ enhanced the tumor suppressive effect of DICB with increased cytotoxic molecules and proliferation of CD8^+^ T cells in MC38 tumor-bearing mice through possibly affecting the gut microbiome.

## Materials and Methods

### Mice

Wild-type C57BL/6 mice at 6 weeks of age were purchased from Jackson Laboratory (USA) and housed under pathogen-free conditions in the Yale University animal facility. Food and water were provided *ad libitum*. Mice in the same treatment group were maintained together in a single cage. All experimental procedures were approved by the Yale University’s Institutional Animal Care and Use Committee (IACUC).

### Cell Culture and Reagents

Murine colon carcinoma MC38 cells were purchased from Kerafast (USA) and cultured at 37°C under 5% CO_2_ in Dulbecco’s Modified Eagle Medium (DMEM) medium supplemented with 10% heat-inactivated fetal bovine serum (FBS) and 100 units/mL penicillin and 100 μg/mL streptomycin.

### Tumor Inoculation and *In Vivo* Treatments

MC38 cells (1x10^5/mouse) were inoculated subcutaneously into the flank of mice. Mice were administered orally with AHCC (18 mg/mouse) or water once a day beginning at day 3 of tumor inoculation with or without intraperitoneal (i.p.) injection of anti-mouse CTLA-4 Ab (50 μg/mouse, clone 9H10, BioXcell) and anti-mouse PD-1 Ab (50 μg/mouse, clone RMP1-14, BioXcell), or isotype control antibodies (BioXcell) twice at 3 day intervals when tumors reached a size of 50 to 100 mm^3^. Tumor size was monitored and measured every 2-3 days with a caliper after the tumor become palpable as previously described (11).

### Antibiotics Treatment

Ampicillin (1 mg/ml), streptomycin (5 mg/ml), and colistin (1 mg/ml) (Sigma-Aldrich) were added in sterile drinking water. Mice were treated with antibiotics starting approximately 3 weeks prior to tumor inoculation and remained on antibiotics until sacrifice. Antibiotics added waters were changed every 4-5 days.

### Flow Cytometric Analysis

Tumors and spleens were harvested from mice and processed into single cell suspension for flow cytometric analysis. Tumors were cut into small pieces and mixed with DMEM containing collagenase IV (Worthington) and DNase I (Sigma-aldrich) followed by incubating at 37°C for 30 min in the gentle MACS dissociator (Miltenyi Biotec). The cell suspension was subsequently passed through a 100 μm nylon filter. Spleens were homogenized by grinding with the microscope slides in a petri dish followed by straining through a 70 μm nylon filter. Red blood cells (RBCs) were lysed with an ACK buffer.

For AHCC^®^ *in vitro* treatment, the splenocytes from wild-type C57BL/6 mice were incubated for 3 days with anti-CD3/CD28 antibodies in the presence or absence of AHCC^®^ (0, 20, and 100 µg/ml). Antibodies for flow cytometry staining were: CD45-BUV395, CD3-APC-Cy7, CD4-Alexa700, CD8-Pacific Blue, PD-1-FITC, Granzyme B-PE, CTLA-4-PE-Cy5, Ki-67-BV605, PD-L1-PE-Cy7, NK1.1-BV605, Ly6C-Pacific Blue, Ly6G-PE-Cy7, CD11b-APC. All antibodies were purchased from BioLegend, BD Biosciences, or Thermofisher Scientific. Non-viable cells were excluded by using Live/Dead fixable aqua dead cell stain kit (Invitrogen). Intracellular staining was performed using a Foxp3/transcription factor staining buffer set (Invitrogen) according to the manufacturer’s protocol. The cells were analyzed on an LSRII flow cytometer (BD Biosciences). Data were analyzed using FlowJo software (FlowJo, LLC).

### 16s rRNA Pyrosequencing and Microbiome Composition qPCR Analysis

Fecal samples were collected on sacrifice day from mice orally administered with water or AHCC^®^ in the presence of DICB treatment. The samples were processed and analyzed at the ZymoBIOMICS service (Zymo research, Irvine, California). DNA extraction was performed, and the DNA samples were prepared for targeted sequencing with the *Quick*-16S NGS library prep kit. For microbiome composition analysis, genomic DNA was isolated from stool samples using the QIAamp Fast DNA Stool Mini Kit (Qiagen) according to the manufacturer’s protocol. qPCR analysis was performed using Mx3005P Real-Time PCR system (Agilent Technologies) and iQ SYBR^®^ Green supermix (Bio-rad). Amplification included an initial denaturation step at 95°C for 10 min followed by 40 cycles of 95°C for 30 sec, 60°C for 1 min, and 72°C for 1 min. The qPCR data were normalized to total bacteria expression levels. The target primers for *Faecalibacterium prausnitzii* and total bacteria were described in previous studies (12).

### Statistical Analysis

GraphPad Prism 8 software was used for conducting statistical analysis. *P* values were obtained using the unpaired student’s *t* test for determining statistical significance. The one-way ANOVA was used to compare tumor volumes in different groups. Values of *P* < 0.05 were considered significant. Data are shown as mean ± SEM.

## Results

### AHCC^®^ Enhanced the Tumor Suppressive Effect of DICB in the Growth of Murine MC38 Tumors

**Figure 1:**
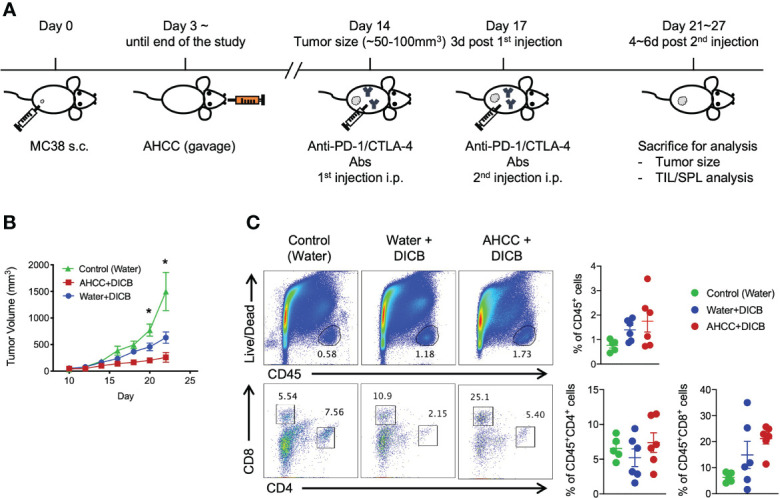
AHCC^®^ enhances the anti-tumor effect of dual PD-1/CTLA-4 blockade in murine MC38 tumors. **(A)** Schematic diagram of AHCC^®^ and dual PD-1/CTLA-4 blockade treatment. Mice were inoculated subcutaneously with 1x10^5^ MC38 tumor cells. Water or AHCC^®^ (18 mg/mouse) administration by oral gavage from 3 days post MC38 tumor inoculation to the end of the experiment. Mice were treated twice at 3-day intervals with dual PD-1/CTLA-4 blockade (50 µg each) when tumors reached a size of approximately 50 to 100 mm^3^. After 4~6 days from the second treatment of the antibodies, mice were sacrificed for further analysis. **(B)** Tumor growth was monitored every other day by measuring tumor volume (mm^3^) (n = 5-6/group, representative data from 3 independent experiments). Lines and error bars indicate mean ± standard error of mean (SEM), respectively. *P* values were determined using the one-way ANOVA with the Dunnett’s multiple comparisons test. **(C)** Flow cytometric analysis of tumor infiltrating CD45^+^ hematopoietic cells, CD8^+^ and CD4^+^ T cells in MC38 tumor-bearing mice treated with dual PD-1/CTLA-4 blockade and water or AHCC^®^. Each dot indicates one mouse. Dots and error bars indicate mean ± standard error of mean (SEM), respectively. Representative data shown from three independent experiments. *P* values were determined using the unpaired Student’s *t*-test *P < 0.05.

We first tested whether AHCC^®^ could suppress the growth of MC38 tumors in mice. Three days after inoculating MC38 tumors, mice were started on oral AHCC^®^ or water. The tumor volumes were similar between the AHCC^®^- and water-treated groups throughout the experiment (**Supplementary Figure 1A**). We analyzed tumor infiltrating CD45^+^ hematopoietic cells including CD4^+^ and CD8^+^ T cells. The frequencies of these cells were similar in mice treated with AHCC^®^ and water (**Supplementary Figure 1B**). The expression levels of the checkpoint inhibitory molecule PD-1, the cell activation marker CTLA-4, the cytotoxic molecule granzyme B, and the cell proliferation marker Ki-67 by tumor infiltrating and splenic CD8^+^ and CD4^+^ T cells were not different between the groups (**Supplementary Figures 1C–F**). We next explored whether AHCC^®^ could enhance the tumor suppressive effect of DICB in MC38 tumor-bearing mice (**Figure 1A**). Mice treated with a combination of AHCC^®^ and DICB had smaller tumor volumes compared to mice treated with a combination of water and DICB (**Figure 1B**) although the frequencies of tumor infiltrating hematopoietic cells (CD45^+^), CD4^+^ and CD8^+^ T cells were similar in mice treated with AHCC^®^ and water in addition to DICB (**Figure 1C**). Both groups also had similar frequencies of natural killer cells, granulocytic (CD11b^+^Ly6G^+^Ly6C^low^) and monocytic (CD11b^+^Ly6G^-^Ly6C^high^) myeloid derived suppressor cells (MDSCs), which have the capacity to inhibit T cell function (13), in the tumors (**Supplementary Figures 2A, B**). These findings support that AHCC^®^ can enhance the tumor suppressive effect of DICB in mice bearing MC38 tumors.

### AHCC^®^ Increased Granzyme B and Ki-67 Expression by Tumor Infiltrating CD8^+^ T Cells in MC38 Tumor-Bearing Mice Treated With DICB Therapy

**Figure 2:**
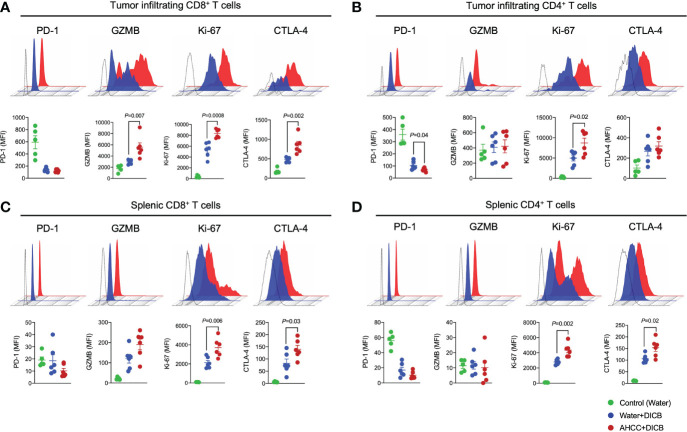
A combination of AHCC^®^ and dual PD-1/CTLA-4 blockade increases granzyme B and Ki-67 expression by tumor-infiltrating CD8^+^ T cells in murine MC38 tumors. Flow cytometric analysis of PD-1, granzyme B (GZMB), Ki-67, and CTLA-4 expression by tumor-infiltrating **(A, B)** and splenic **(C, D)** CD8^+^ and CD4^+^ T cells in MC38 tumor bearing mice treated with DICB and AHCC^®^ or water as in **Figure 1A**. Each dot indicates one mouse (n = 5-6/group, representative data from 3 independent experiments). Lines and error bars indicate mean ± standard error of mean (SEM), respectively. MFI, mean fluorescent intensity. Representative data shown from three independent experiments. *P* values were determined by the unpaired Student’s *t-*test.

Anti-tumor T cell immunity induced by DICB therapy is critical for controlling malignancies. We thus analyzed the characteristics of tumor infiltrating T cells to determine the possible effect of AHCC^®^ and DICB on these cells in MC38 tumor-bearing mice. Mice treated with the combination of AHCC^®^ and DICB had increased expression of the cytotoxic molecule granzyme B, the cell proliferation marker Ki-67, and the cell activation marker CTLA-4 by tumor infiltrating CD8^+^ T cells compared to mice treated with water and DICB (**Figure 2A**). Our finding of the increased CTLA-4 expression by CD8^+^ T cells, which likely reflects enhanced T cell activation, is in line with the results of a previous study reporting increased CTLA-4 expression by peripheral T cells, including CD8^+^ T cells, in patients with non-small cell lung cancer who received a combination of chemotherapy and anti-CTLA-4 ipilimumab (14). The expression levels of PD-1 by tumor infiltrating CD8^+^ T cells were similar between the two treatment groups. We also analyzed the expression of the same molecules by tumor infiltrating CD4^+^ T cells. Mice treated with AHCC^®^ and DICB had increased expression of Ki-67 and decreased expression of PD-1 by tumor infiltrating CD4^+^ T cells compared to mice treated with water and DICB (**Figure 2B**). Adding AHCC^®^ to DICB did not affect the expression levels of PD-L1 and Ki-67 by non-hematopoietic cells in the tumors (**Supplementary Figure 2C**). Similarly to tumor-infiltrating T cells, splenic CD8^+^ and CD4^+^ T cells in mice treated with the combination of AHCC^®^ and DICB had increased expression of Ki-67 and CTLA-4 while granzyme B expression in splenic CD8^+^ and CD4^+^ T cells were barely detected by flow cytometry (**Figures 2C, D**). These findings suggest that the enhanced tumor suppressive effect of a combination of AHCC^®^ and DICB is related in part to increased activation, proliferation and cytotoxicity of T cells, especially CD8^+^ T cells, in tumors and spleen.

### Gut Microbiome Altered in MC38 Tumor-Bearing Mice Treated With the Combination of AHCC^®^ and DICB Therapy

**Figure 3:**
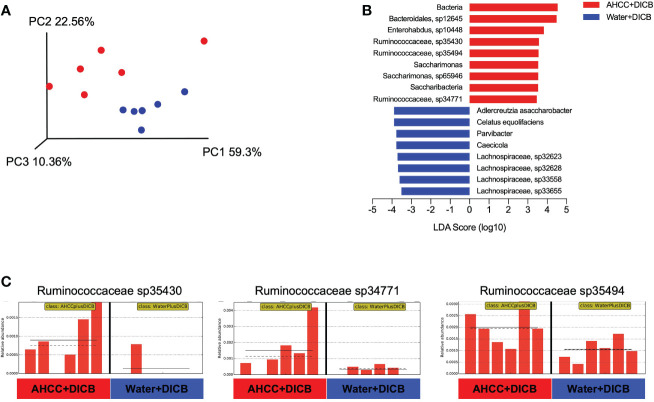
AHCC^®^ alters the gut microbiome in MC38 tumor-bearing mice treated with dual PD-1/CTLA-4 blockade. Gut microbiome analysis was done in MC38 tumor-bearing mice treated with AHCC^®^ or water in the presence of DICB therapy by sequencing bacterial 16s rRNA genes in the feces collected on the sacrificing day. **(A)** Principal coordinate analysis (PCoA) plot of bacterial β-diversity based on Unweighted Unifrac Plots of unique amplicon sequence variants (ASV) (n = 6/group). **(B)** A linear discriminant analysis (LDA) combined with effect size measurements (LEfSe) showing significant bacterial differences in the two groups (n = 6/group). Logarithmic LDA score threshold > 3.0 and *P* value of < 0.05 were considered significant. **(C)** Bar graphs showing the abundance distribution profile of indicated *Ruminococcaceae* species in the two groups (n = 6/group). The horizontal and dotted lines in the panels indicate the group mean and median, respectively.

Previous studies showed the possible relationship of the gut microbiome with cancer immunotherapy in animal models and humans (15). We thus investigated whether adding AHCC^®^ to DICB therapy altered the gut microbiome by sequencing bacterial 16s rRNA genes in the feces collected on sacrificing day from MC38 tumor-bearing mice treated with AHCC^®^ or water in the presence of DICB therapy. Both groups had similar levels in the abundance of bacterial gene and genome copies as well as in the number of observed species and Shannon diversity index as determined by alpha diversity which is a measurement of the microbial diversity of each sample (**Supplementary Figures 3A, B**). However, in assessing beta diversity which is an estimate of dissimilarity between groups using Unweighted Unifrac dissimilarity of unique amplicon sequence variants (ASV), most samples were clustered based on treatment types as shown in a principal coordinated analysis plot (**Figure 3A**). We next employed a linear discriminant analysis (LDA) effect size (LEfSe) method to explore taxa whose distributions are significantly different between the groups. This analysis revealed the species in *Ruminococcaceae* family and *Saccharimonas* were enriched significantly (LDA score >3, *P* < 0.05) in mice treated with a combination of AHCC^®^ and DICB (**Figures 3B, C**). Of note, bacterial taxa within the *Ruminococaceae* family were reported to be associated with the better clinical response to cancer immunotherapy (15), suggesting the possible implication of gut microbiome in promoting the therapeutic effect of DICB by adding AHCC^®^ in MC38 tumor-bearing mice.

### Antibiotics Treatment Abrogates the Additive Therapeutic Effect of AHCC^®^ to DICB Therapy in MC38 Tumor-Bearing Mice

**Figure 4:**
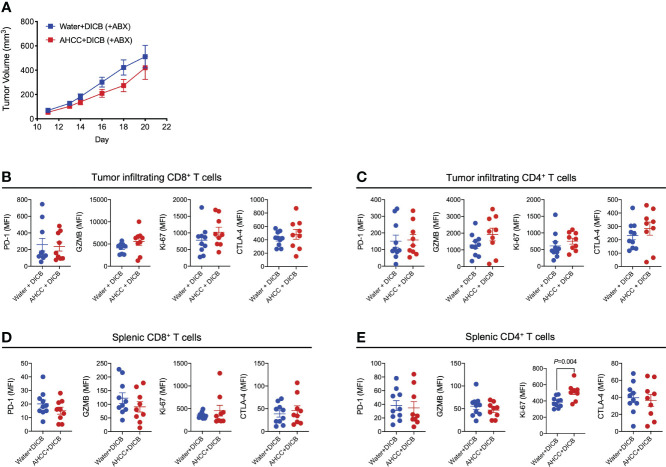
Administration of antibiotics abrogates the additive anti-tumor effect of AHCC^®^ in MC38 tumor-bearing mice treated with dual PD-1/CTLA-4 blockade. Mice were treated for approximately 3 weeks prior to subcutaneous MC38 tumor inoculation with a combination of 3 antibiotics (ampicillin, streptomycin, and colistin) by adding the drugs to drinking water and continued until sacrifice. As described in **Figure 1A**, tumor bearing mice were subsequently treated with water or AHCC^®^ in addition to dual PD-1/CTLA-4 blockade i.p. injection (50 µg each, twice at 3-day intervals). **(A)** Tumor growth was determined by measuring tumor volume (mm^3^). Flow cytometric analysis of PD-1, granzyme B (GZMB), Ki-67, and CTLA-4 expression (mean fluorescent intensity or MFI) by tumor-infiltrating **(B, C)** and splenic **(D, E)** CD8^+^ and CD4^+^ T cells. Each dot indicates one mouse (n =9-10/group). Lines and error bars indicate mean ± standard error of mean (SEM), respectively. Data from two independent experiments. *P* values were determined using the unpaired Student’s *t*-test.

We further explored the possible implication of the gut microbiome in the combination therapy of AHCC^®^ and DICB in MC38 tumor-bearing mice by administrating antibiotics. Mice were pretreated for 2-3 weeks prior to tumor inoculation with a combination of ampicillin, streptomycin, and colistin, which were continued until the end of the experiment. Although there was a trend towards the decreased tumor volume in mice treated with the combination of AHCC^®^ and DICB compared to mice treated with water and DICB, this difference was not statistically significant (**Figure 4A**). Similar changes were also observed in analyzing granzyme B and Ki-67 expression by tumor infiltrating and splenic CD8^+^ and CD4^+^ T cells except Ki-67 expression in splenic CD4^+^ T cells (**Figures 4B–E**). These findings support the implication of the gut microbiome in mediating the combinational effect of AHCC^®^ and DICB therapy.

## Discussion

The introduction of antibodies targeting immune checkpoint molecules in oncology has revolutionized anti-cancer therapy. Monoclonal antibodies blocking the binding of the immune checkpoint molecules PD-1 and CTLA-4 to the ligands can restore exhausted T cell immunity against tumors, leading to the suppression of tumor growth. Natural products such as edible mushrooms have been considered to have possible benefits in treating diseases including cancers although therapeutic benefits and mechanisms of most natural products remain to be shown (5). Polysaccharides in mushrooms are suggested to have immune boosting effects by affecting immune cells including T cells (7). Here we investigated the hypothesis that AHCC^®^ could increase the anti-tumor effect of DICB *via* enhancing T cell immunity using a heterotopic murine colon cancer model. The results of our study showed that AHCC^®^ enhanced the tumor suppressive effect of DICB as well as cytotoxic molecule expression and proliferative capacity of T cells, especially tumor infiltrating CD8^+^ T cells, in MC38 tumor-bearing mice through possibly affecting the gut microbiota.

The potential anti-tumor effect of AHCC^®^ was reported by previous studies using animal models. Mice pre-treated with AHCC^®^ had delayed tumor growth after B16 melanoma inoculation compared to mice pre-treated with water (9). In hepatoma 22 tumor-bearing mice, the anti-tumor effect of low-dose 5-fluorouracil (5-FU) was enhanced by AHCC^®^ (10). We first explored whether AHCC^®^ alone could suppress the growth of MC38 tumor in mice. The levels of tumor growth were similar in mice treated with AHCC^®^ and water, suggesting that AHCC^®^ alone may not be sufficient in controlling MC38 tumor growth. A previous study reported that the partial tumor suppressive effect of anti-PD-1 or -CTLA-4 antibody monotherapy in MC38 tumor-bearing mice which received 4 doses of 200 μg/injection over 10 days (16). A combination of anti-PD-1 and CTLA-4 at the same dose had a greater anti-tumor effect against MC38 tumor in the same study. In our study, we found a tumor suppressive effect of a combination of low dose anti-PD-1 and -CTLA-4 antibodies (100 μg/injection x 3 for each antibody, 3 days apart) in the same mouse model. Of note, the frequency of immune-related adverse events is associated with the drugs used, exposure time, and the dose administered (17). Considering these points, we explored the additive effect of AHCC^®^ to the combinational treatment of low dose anti-PD-1 and -CTLA-4 antibodies (for each antibody 50 μg/injection x 2, 3 days apart). The results of our study showed that MC38 tumor-bearing mice treated with AHCC^®^ and DICB had smaller tumor size and less tumor growth compared to the same tumor-bearing mice treated with water and DICB. These findings support the possible consideration of testing the implication of AHCC^®^ in treating human cancers along with immune checkpoint blockade. Similar approaches of combining immune checkpoint blockade, including dual blockade, with tumor vaccines, especially dendritic cell (DC)-based, immunoadjuvant nanocomplexes, or chemotherapeutic agents were shown to be effective in murine cancer models (18–23). DCs that help T cell activation by presenting antigen and providing co-simulation to T cells can have a critical role in tumor immunity (24, 25). The possible effects of AHCC^®^ on DCs, especially myeloid DCs (mDC), were previously reported in healthy people (26, 27), raising the possible consideration of future studies investigating the effects of AHCC^®^ with or without immune checkpoint blockade on DCs in the context of tumor microenvironments.

The interface of the immune system with the gut microbiota is critical for the tolerance of commensal bacteria and food allergens as well as for host defense against the invasion of pathogenic bacteria. In addition, the gut microbiota can participate in the development and regulation of systemic immune responses by affecting innate and adaptive immune cells (28). Indeed, an accumulating body of evidence from animal and human studies supports the possible relationship of the gut microbiome with cancer immunotherapy by affecting its efficacy and toxicities (15). The optimal anti-tumor effects of anti-CTLA-4 antibody therapy required specific Bacteroides species including *Bacteroides thetaiotaomicron* and *Bacteroides fragilis* in mice and humans with melanoma (29). Similarly, *Bifidobacterium* species enhanced the efficacy of anti-PD-L1 antibody with increased dendritic cell maturation and CD8^+^ T cell activity in the tumor microenvironment in a murine melanoma model (30). Analysis of fecal microbiome samples in melanoma patients treated with anti-PD-1 therapy showed an increase in the relative abundance of the *Ruminococcaceae* family in treatment responding patients (31). *Ruminococcaceae spp* were also enriched in patients with epithelial tumors on PD-1-based immunotherapy who had progression-free survival (PFS) longer than 3 months compared to those with PFS shorter than 3 months (32). These studies support the possible implication of the *Ruminococcaceae* family in the gut in enhancing the efficacy of anti-cancer immunotherapy. In our study, the relative abundance of the species in *Ruminococcaceae* family increased in MC38 tumor-bearing mice treated with AHCC^®^ and DICB compared to the same mice treated with water and DICB. To further support this finding, we analyzed *Faecalibacterium prausnitzii* which is a leading representative of the phylum *Firmicutes*, class *Clostridium*, family *Ruminococcaceae* and the most dominant species within the *Ruminococcaceae* family using a qPCR assay (12). The results of this analysis showed increased abundance of *F. prausnitzii* in the stool samples from the mice treated with AHCC^®^ and DICB compared to water and DICB. In addition, administrating antibiotics abrogated the benefit of adding AHCC^®^ to DICB therapy in these mice. These findings suggest the possible role of the gut microbiota in moderating the beneficial effect of AHCC^®^ in the setting of DICB therapy. We believe future studies identifying specific bacteria related to the combinational effect of AHCC^®^ and DICB therapy and administrating such bacteria to tumor bearing mice would be warranted to address this point.

The combinational effect of AHCC^®^ and DICB therapy on tumor could be mediated by enhanced cytotoxicity and proliferation capacity of tumor infiltrating T cells as noticed by increased expression of granzyme B and Ki-67 in these cells in mice treated with AHCC^®^ and DICB therapy. Although such effect can be through affecting the interface of the gut microbiota and the immune system, AHCC^®^ could have a direct effect on T cell activation. Indeed, we noticed increased expression of Ki-67 by CD4^+^ and CD8^+^ T cells and granzyme B by CD4^+^ T cells in the presence of T cell stimulation with anti-CD3 and CD28 antibodies *in vitro* (**Supplementary Figure 5**). The cytotoxic molecule granzyme B is highly expressed by T cells, especially CD8^+^ T cells. Immunohistochemical staining and *in vivo* imaging, using positron emission tomography (PET), of intratumoral granzyme B may serve as a predictive biomarker for PD-1 blockade in melanoma (33). Also, in animal models of cancers including MC38 colon cancer, intratumoral granzyme B quantification by PET imaging was a highly sensitive and specific early measure of therapeutic efficacy for checkpoint inhibitor regimens (34). We analyzed Ki-67 to assess T cell proliferative capacity in tumors using flow cytometry. A previous study reported increased expression of Ki-67 by CD4^+^ and CD8^+^ T cells in patients with melanoma who received anti-CTLA-4 ipilimumab (35). Ki-67^+^CD8^+^ T cells expressing IFN-γ correlated with survival benefit in patients with hepatocellular carcinoma treated with sorafenib, an ERK inhibitor (36). In MCA205 tumor-bearing mice, anti-OX40 agonistic antibody treatment, which activates T cells, resulted in increased Ki67^+^CD8^+^ T cells in the tumors (37). Overall, these findings support the possible biological implication of our results showing increased Ki-67 and granzyme B expression by tumor infiltrating CD4^+^ and CD8^+^ T cells in mice treated with a combination of AHCC^®^ and DICB therapy.

In summary, we showed that AHCC^®^ promoted the tumor suppressive effect of dual PD-1 and CTLA-4 blockade in mice bearing MC38 colon cancer through possibly affecting the gut microbiome. This additive effect was accompanied by increased expression of the cytotoxic molecule granzyme B and the cell proliferation molecule Ki-67 in tumor-infiltrating T cells, especially CD8^+^ T cells. Although additional mechanistic studies are warranted, these results introduce a concept of combining immune checkpoint blockade with natural products such as AHCC^®^ in cancer treatment.

## Data Availability Statement

The original contributions presented in the study are publicly available. This data can be found here: https://www.ncbi.nlm.nih.gov/bioproject/PRJNA807301.

## Ethics Statement

The animal study was reviewed and approved by Yale University’s Institutional Animal Care and Use Committee (IACUC).

## Author Contributions

HJP, MS, IK, TT, JT, and KH conceptualized the study. HJP, SB, IP, MS, and IK conducted the experiment. HJP and IK performed the analysis. HJP, IK, TT, JT and KH participated in writing the manuscript. All authors reviewed the manuscript and approved the submitted version.

## Funding

This study received funding from Amino Up Co., Ltd, Sapporo, Japan.

## Conflict of Interest

IK received research funding from Amino Up Co., Ltd, Sapporo, Japan, the manufacturer of AHCC^®^ that was studied in this work and is a consultant of Amino Up Co., Ltd. TT, JT, and KH are employees of Amino Up Co., Ltd. The funder had the following involvement with the study: experimental design and manuscript writing.

The remaining authors declare that the research was conducted in the absence of any commercial or financial relationships that could be construed as a potential conflict of interest.

## Publisher’s Note

All claims expressed in this article are solely those of the authors and do not necessarily represent those of their affiliated organizations, or those of the publisher, the editors and the reviewers. Any product that may be evaluated in this article, or claim that may be made by its manufacturer, is not guaranteed or endorsed by the publisher.
