# Supplementary material for: AHCC®, a Standardized Extract of Cultured Lentinula Edodes Mycelia, Promotes the Anti-Tumor Effect of Dual Immune Checkpoint Blockade Effect in Murine Colon Cancer
Source: Front Immunol. 2022 Apr 20;13:875872. doi: 10.3389/fimmu.2022.875872 (PMC9066372; doi:10.3389/fimmu.2022.875872)
Supplement: Supplementary file 1 [file DataSheet_1.docx]

**Supplementary Figure**

**Supplementary Figure 1** MC38 tumor growth was not suppressed by AHCC® in mice. **(A)** Mice were inoculated subcutaneously with 1x10^5^ MC38 tumor cells and treated with water or AHCC® (18 mg/mouse) by oral gavage from 3 days post MC38 tumor inoculation to the end of the experiment. Tumor growth was monitored every other day by measuring tumor volume (mm^3^). **(B)** Flow cytometric analysis of tumor infiltrating CD45^+^ hematopoietic cells, CD8^+^ and CD4^+^ T cells in MC38 tumor-bearing mice treated with water or AHCC®. **(C-F)** Scatter graphs showing the mean fluorescent intensity (MFI) of PD-1, granzyme B (GZMB), Ki-67, and CTLA-4 expression by tumor-infiltrating and splenic CD8^+^ and CD4^+^ T cells as determined by flow cytometry. Each dot indicates one mouse. Lines and error bars indicate mean ± standard error of mean (SEM), respectively. Representative data shown from two independent experiments. *P* values were determined by the unpaired Student’s *t-*test.

**
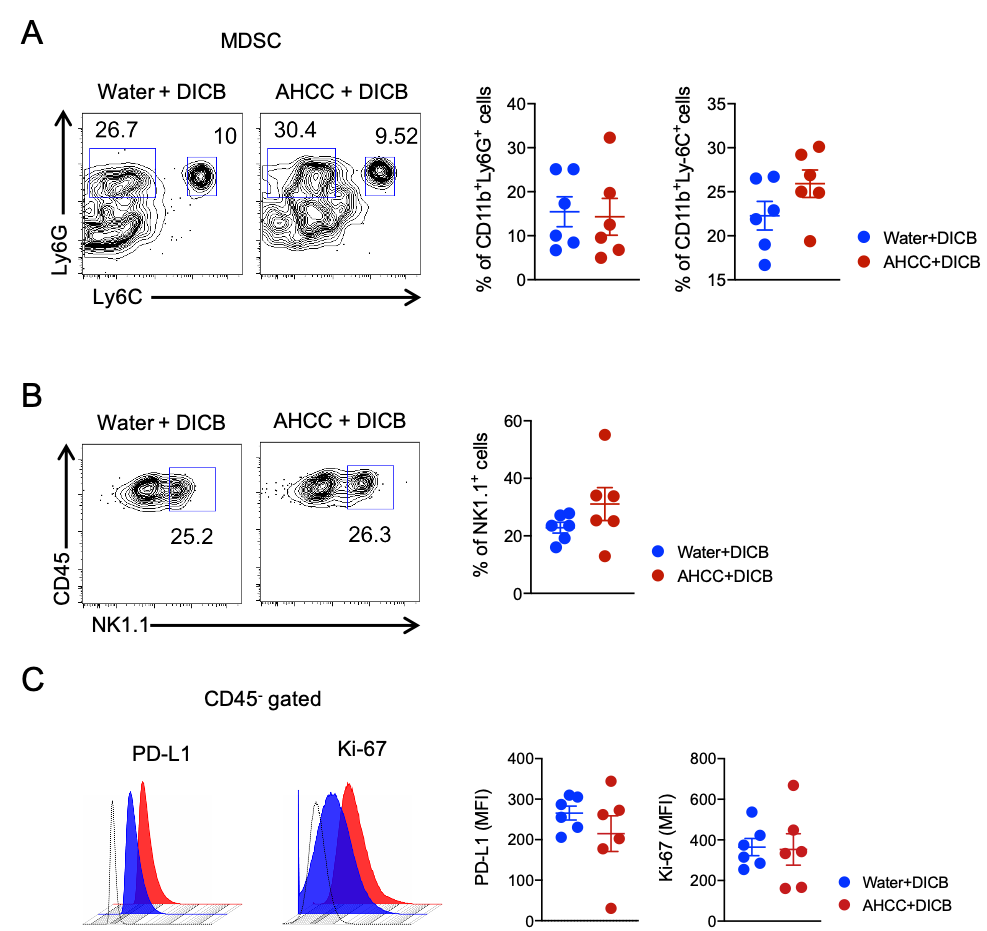
**

**Supplementary Figure 2** A combination of AHCC® and DICB did not affect the frequency of myeloid derived suppressor cells (MDSCs), natural killer (NK) cells, and non-hematopoietic cells in murine MC38 tumor. The frequencies of MDSCs (CD11b^+^Ly6G^+^ and CD11b^+^Ly6C^+^) (**A**) and NK cells (**B**) in MC38 tumors of mice treated with DICB in combination with water or AHCC® as described in Figure 1. **(C)** PD-L1 and Ki-67 expression (MFI) by CD45^-^ non-hematopoietic cells in MC38 tumors from mice treated with AHCC® or water in the presence of DICB. Representative data shown from three independent experiments. *P* value was determined by the unpaired Student’s *t* test.


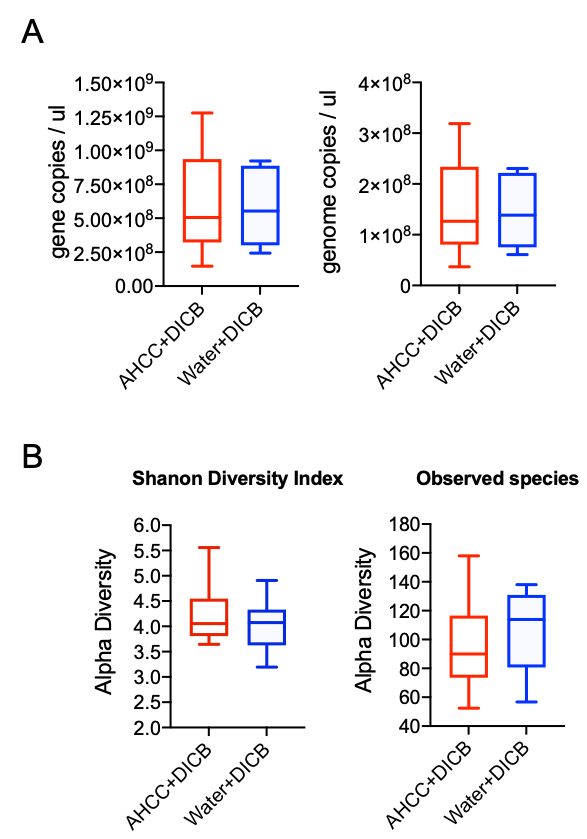


**Supplementary Figure 3** A combination of AHCC® and DICB had no effects on the absolute abundance and alpha diversity of gut microbiome. **(A)** The absolute abundance of gene and genome copies per microliter in stool samples from MC38 tumor bearing mice treated with AHCC® or water in the presence of DICB as described in Figure 1. **(B)** Alpha diversity was analyzed, and box-and whisker plots show sample evenness (Shannon Diversity index) and richness (observed species) in stool samples.

**Supplementary Figure 4** A combination of AHCC® and DICB increased abundance of *Faecalibacterium prausnitzii*, the most dominant species within the *Ruminococcaceae* family. QPCR analysis was performed targeting *Faecalibacterium prausnitzii*, which is a leading representative of the phylum *Firmicutes*, class *Clostridium*, family *Ruminococcaceae,* in the stool samples from the mice treated with water or AHCC® in the presence of DICB. Representative data shown from two independent experiments. *P* value was determined by the unpaired Student’s *t* test.

**Supplementary Figure 5.** *In vitro* effects of AHCC® on granzyme B and Ki-67 expression by CD4^+^ and CD8^+^ T cells stimulated with anti-CD3/CD28 antibodies. Splenocytes from wild-type C57BL/6 mice were incubated for 3 days with anti-CD3/CD28 antibodies in the absence or presence of AHCC® (0, 20, and 100 µg/ml). Flow cytometric analysis of splenic CD8^+^ and CD4^+^ T cells gated on CD62L^-^CD44^high^ effector memory T cells. **(A-F)** Scatter graphs showing the mean fluorescent intensity (MFI) of PD-1, granzyme B (GZMB), and Ki-67 expression by splenic CD8^+^ and CD4^+^ T cells. Each dot indicates one mouse. Lines and error bars indicate mean ± standard error of mean (SEM), respectively. *P* values were determined by the unpaired Student’s *t-*test.
